# Global health activists’ lessons on building social movements for Health for All

**DOI:** 10.1186/s12939-020-01232-1

**Published:** 2020-07-06

**Authors:** Connie Musolino, Fran Baum, Toby Freeman, Ronald Labonté, Chiara Bodini, David Sanders

**Affiliations:** 1 Southgate Institute for Health, Society, and Equity, Flinders University, GPO Box 2100, Adelaide, SA 5001 Australia; 2 School of Epidemiology and Public Health, University of Ottawa, 600 Peter Morand Crescent, Ontario, Ottawa K1G 5Z3 Canada; 3 Centre for International and Intercultural Health (CSI), University of Bologna, Bologna, Italy; 4 School of Public Health, University of the Western Cape, Cape Town, South Africa

**Keywords:** Civil society, Health activism, People’s health Movement, Neoliberalism, Health for all, Health equity, Social determinants of health

## Abstract

**Background:**

The People’s Health Movement (PHM) was formed in 2000 and drew inspiration from the Alma Ata Declaration on Primary Health Care’s ‘Health for All’ (1978). Since then PHM has been an active part of a global counter-hegemonic social movement. This study aimed to gain insights on social movement building, drawing on the successes and failures reported by activists over their experiences of working in the Health for All social movement to improve health, justice and equity.

**Methods:**

Qualitative research methods were employed in this study to capture complex and historical narratives of individual activists, through semi-structured interviews and subsequent thematic analysis of transcripts. The research design and analysis were informed by social movement theory and literature on health activism as a pathway for social change. In this study we examine the semi-structured interviews of 15 health activists who are part of the PHM, with the aim of deriving lessons for strengthening movements for Health for All.

**Results:**

This study locates the activists’ narratives within a socio-political analysis of the global trends of late modern individualism and capitalist neoliberalism. This highlights the challenges faced by civil society groups mobilising collective action and building social movements for Health for All. The study found that within the constraints of the neoliberal socio-political and economic conditions which have caused the rise in social and health inequities, this group of long-term health activists have been nurturing alternative approaches to structuring society and building collective agency to improve health.

**Conclusion:**

The practical long-term experiences of the PHM activists examined in this study contribute to a better understanding of the processes and motivations that lead to and sustain health activism, and the dilemmas, strategies, impacts and achievements of such activism.

## Background

Civil society activism can lead to social change to promote health equity through multiple pathways including advocating for policy change, program implementation, community development and the building of social movements [1–3]. In this study we analyse the narratives of 15 civil society health activists who are part of the People’s Health Movement (PHM) to understand better the processes and motivations that lead to health activism, and the dilemmas, strategies, impacts and achievements of such activism.

Civil society is generally considered to embrace those social relations and formations that exist outside of state and market relations, and through which people voluntarily undertake collective action for normative and substantive purposes [4, 5]. Civil society activism varies across countries, and navigating state, governing and funding bodies and other vested interests can be a tenuous path for many groups [6].

Health activism is defined in this study as efforts aimed at improving population health and challenging health inequities, whether directed at health systems or at social, economic, political, and environmental determinants of health and which largely sits outside activists’ core employment activities [5, 6]. Our study follows on from Ollis’ earlier work on civil society activism, which underscored the importance of reflecting upon ‘the embodied pedagogy of activists who gain knowledge through the practical experience of being in the world of activism’ [7].

The PHM is a global network of activist organisations formed in December 2000 in response to the failure to achieve Health for All by the Year 2000, a goal set in the 1978 Alma Ata Declaration of Primary Health Care [8]. Numerous studies attribute failure of the original HFA goal to several factors. These include: the rise of neoliberal economics and retrenchment of state-funded programs, the imposition of structural adjustment programs in many parts of the world, and increased privatisation of formerly public goods and services. Furthermore, the eclipse of Alma-Ata’s more comprehensive vision of health system reform by one that focused on ‘selective’ disease-focused interventions often chosen by donor agencies or countries undermined local capacity [9–17].

At the same time, civil society opposition to these global macroeconomic and political trends, and some countries’ efforts to withstand them by retaining progressive elements in their health and social policies [18], lent support to a renewal of activism to ‘revitalize’ the visionary HFA goal. From its foundation, the PHM has consistently argued that ‘the struggle for health is a political struggle’, one ‘which challenges the fundamental practices of our society and the trends which shape them’ [19].

Our study aimed to provide critical insights on strategies used to build and sustain civil society health activism. Such activism is vital at a time when the goal of Health for All is receding in the face of the economic and ecological problems, including the COVID-19 crisis and life expectancy is declining in some parts of the world [20]. The aims of the study are to contribute to understanding how civil society can create social change to improve health, and to derive transferable lessons for strengthening social movements for health and health equity.

## Methods

This study is part of a larger 4 year multi-centre research project undertaken between 2014 and 2018 through the PHM and supported by the Canadian International Development Research Centre (IDRC) to explore civil society engagement in the struggle for Health for All. The aim of the research was to better understand civil society engagement on health, with the goal of improving PHM’s and others activist practice. This component of the research examined the narratives of long-term PHM civil society health activists and the findings fed into the main project’s final report and recommendations [21].

### Participants

This study examined the experiences of 15 participants who were involved in civil society activism through the People’s Health Movement. The PHM is a global network of activists and therefore participants were located in different countries across the world at the time of data collection (including South Africa, Australia, India, UK, USA, Belgium, Italy, Philippines, Vietnam, Nicaragua and Brazil), representing the diversity of the PHM network and the global relevance of the HFA goal. The participants reported being involved in health activism for all their adult lives, which ranged between 20 to 70 years. The purpose for recruiting activists with long-term experience was to capture their historical knowledge and analysis of the changing socio-political landscape in which PHM operates and to reflect on failures and successes over their personal activist careers and the social movements they have been involved in. Eight of the participants were men and seven were women.

### Recruitment

A purposive sample of health activists was recruited through PHM networks. The inclusion criteria were intentionally broad, including participants who were active in the PHM for over 10 years. The participants were selected on the basis of their formal roles within PHM in either the global secretariat or governance structures rather than because of their country of residence. The recruitment process was aided by the research team being involved in the PHM. While the authors acknowledge this may present a bias, it also contributed to the trust between the interviewer and participant and allowed for a deeper sharing of personal stories. All participants approached agreed to be interviewed. A narrative guide containing 20 open ended questions was developed through email consultation with the PHM Steering Council, and included questions such as ‘What lessons have you learnt through the process of activism?’ and ‘What national/international policy issues have you worked on?’. The guide was sent to all participants along with a participant information sheet and consent form before the interview. Informed consent to participate in the study was obtained from all participants.

The study received ethics approval by the Senate Research Committee of the University of Western Cape and Flinders University’s Social and Behavioural Research Ethics Committee.

### Data collection

Seven researchers including authors 1, 2 and 6 conducted the 15 semi-structured interviews that were completed from January 2016 to October 2017. The interviews were conducted in a number of locations and through different modes, both in person (in office meeting rooms and at PHM events), through skype and by phone. This enabled the project to include activists in multiple countries.

All interviews were conducted in English, in which participants were highly proficient. Interviews varied in length (between 1 to 3 h) and were recorded and professionally transcribed. Participants were given the option of reading and commenting on their interview transcripts, and have been given pseudonyms to protect their identity.

### Analytical approach

Our analytic framework followed an inductive thematic approach, commonly used in social science research to identify major themes in textual data. This approach allows themes to emerge directly from the data, not from pre-conceived concepts, and prioritises participants’ experiences and perspectives [22, 23]. Preliminary analysis of the interview transcripts involved open coding with author 1 reading the transcripts line by line to identify and develop ideas, themes and issues from the data, followed by a research team meeting in which general themes were drawn out. A coding framework was then developed and informed by social movement theory and literature on health activism as a pathway for social change [24–26]. The transcripts were coded using the software NVivo 11. One transcript was double coded to ensure research rigour and analytical continuity before commencing coding [27]. The research team found that participants’ reflections on health activism pivoted around dialectic tensions in their activist roles. For example, these included whether civil society activities should be ‘reactive’ or ‘proactive’. Examining these tensions helped to tease out the lessons for health activism, which are presented under the main thematic categories in the Results section: movement building; capacity building; campaigning, advocacy policy dialogue and engaging in governance; and knowledge generation and dissemination. Another team meeting and layer of analysis followed, in which the key arguments presented in the discussion section developed through examining the lessons from the health activists’ narratives, critically in relation to the wider literature and theoretical concepts on social movements, activism, health inequities, neoliberalism and individualism, which formed the central arguments for this paper.

The 15 interviews provided a wealth of knowledge that answered the research questions. However, while there were patterns in the activists’ experiences which we drew together in the coding framework, there were also great diversity in their stories which we could not fully capture in this one paper.

## Results

The activists reported being involved in campaigns in over 40 different policy areas. Some of the extensive and diverse policy areas included: refugee health and rural health, water and sanitation, access to medicines and the Sustainable Development Goals. Similarly, the types of communities, groups and organisations activists reported working with was vast, including: professional groups such as Aboriginal health workers and the academic community; national and international organisations such as trade unions and civil rights movements; community based organisations such as community health centres and an array of grassroots activists; and groups of people, such as women’s groups and people with a disability.^1^

Our findings are structured around four broad themes based on the planning framework devised by the PHM and the Canadian International Development Research Centre (IDRC) research collaboration. The themes represent civil society health activists’ experiences in:

- Movement building
- Capacity building
- Campaigning, advocacy policy dialogue and engaging in governance
- Knowledge generation and dissemination

There is considerable overlap between the themes as they inform one another in multiple ways.

### Movement building

#### Pathways to activism

The activists’ histories indicated that there are many varied pathways to becoming politically active. Some began as health care providers or health promoters, which exposed them to the detrimental impact of inequitable social determinants on people’s health and influenced their decision to become activists alongside their clinical work. Nicolas, for example, grew up in Chile and became politically active while at medical school in the 1960s, stating, ‘I knew that we were not solving the problems in the hospital’. This interplay between delivering health services and working to address structural issues is prominent in the interviewees’ pathways to activism and throughout their careers. For others, activism was a response to living in oppressive regimes or living through war, such as Kate’s experiences protesting against apartheid in South Africa, and Mary’s role as a nurse during World War Two in the USA which steered her into community education and advocacy work. Rohit graduated from medical college in 1971, when 9 million Pakistani refugees crossed the border into India. He worked in government camps delivering health services to the refugees and this experience informed his decision to establish an advocacy network for medical professionals. Four of the activists grew up in socially and politically aware families, such as Angela who was encouraged from a young age to be active in her community and so joined the Scouts, the Red Cross lifeguard and volunteered in a hospital, and Lila who described her mother instilling in her a Gandhian philosophy. Others came to activism through insights gained from formal education and awareness of injustice.

#### Social movements have long histories and deep roots

Interviewees had extensive experience in civil society and reflected upon processes of movement building. A frequent comment was that social movements typically have deep historical roots, with new movements, such as PHM, often building on the achievements and momentum of existing, older ones.

Activists from Central and Latin America and Africa, for example, recounted their involvement in earlier national liberation movements during the 1970s, ‘80s and ‘90s. While working in health promotion in Central America under a dictatorial regime, Angela became involved in organising networks of health activists within and outside of Central and Latin America. She explained how these networks and the knowledge gained through such struggles subsequently informed PHM development in Latin America. In South Africa, Kate described how HFA advocacy was bound up with the history of the ‘politics of the country and the liberation struggle’. The apartheid system necessitated health activists to establish alternative health structures to treat non-whites. After the apartheid regime ended in 1994, health activism shifted to an emphasis on primary health care advocacy, including recognition of the important role played by community health workers.

#### Effective strategies for movement building

Most activists in this study drew on theories of organising (for example one activist cited Melucci’s theory of collective action [28]) and were reflective about how mass movements start. Irene argued that mass mobilisation builds the power of the collective and leads to cultural change:

I think it’s important to create links to create a critical mass of people to bring about change … I don’t think the only way is to be politically exposed or active. But I feel that what is missing now is bigger groups of people mobilising, alliances, networks that are able to make an impact, to aggregate power.

Nicolas argued that, while mass mobilisation is important, a small, vanguard group is necessary for sustained action. He provides a recent example:

Look at the Wall Street [Occupy] movement … it fizzled out … Look at what the Spring Revolution brought us in the Middle East … when the people have had enough the whole thing explodes and you have a semi revolutionary or very active participation of people that is growing, gains visibility in the media and whatever, but at that time you have to find an avant-garde which is capable of sort of sitting aside and looking at potential [for change].

Others spoke of the need for a plurality of activist strategies that contribute to movement building, ranging from street protests, to organised lobbying efforts, and to global policy advocacy projects.

#### Civil society organisations – building social movements or businesses?

Four activists drew a distinction between being part of a movement from participating in the activities of non-governmental organisations (NGOs). They felt being an activist reflected a personal commitment above and beyond any professional or institutional role. The difference between the two was portrayed.

I mean at the moment if we look at civil society in (name of country) there’s a lot of what (name of fellow activist) calls ‘BONGOs’ which are ‘briefcase only NGOs’ and basically where you can feel quite out of sorts because people are working for NGOs that have got no activist background whatsoever … they are there because they can get an extremely good salary. (Kate).

Emma described how in the Philippines in the 1990s ‘NGOs were co-opted or used for legitimising the government’. This is one consequence of a depoliticised health service and NGO workforce. Samy argues another consequence is that the international NGO workforce has become ‘professional lobbyists’ who often fail to appropriately report back to the communities they represent. Indra described activism as ‘mundane’ and ‘nitty, gritty, hard work’ which may not appeal to those who lack the ideological motivation, stating: ‘if you’re looking for glamour, join a corporate bank’.

Although these sentiments distinguish between ‘activism’ and the programmatic work of NGOs, PHM activists also acknowledged that, in the current civil society environment, lines are often blurred between the interests of civil society, and those of the state, international NGOs, and funding bodies.

#### Resources for movement building: health is political, and this should not scare funders

Movement building is hindered by a lack of resources and funding. Study participants noted that funders and policy makers are frequently reluctant to support more progressive groups because they are seen as overly political. As Angela noted:

My organisation has a financial crisis, but so does almost every other organisation because of neoliberal policies and people don’t want to give money to troublemakers, right? So, it’s very hard to get support.

Our activist narratives indicate that health is inevitably political. To give one example:

I remember being invited to South Africa in the 1990s, such an exciting period, you know, apartheid had fallen, they were full of enthusiasm for new primary health care policies, but over the years having watched how that promise hasn’t been entirely fulfilled and realising how intimately health policy is tied up with politics. (Elaine).

The activists also spoke about the need for funders to be prepared to fund groups who do advocacy. As one participant argued:

From about after 2000 through ‘til probably the global financial crisis, we did have organisations, particularly in Europe, who would fund [PHM] and we could have quite a strong secretariat. But that’s been whittled away now and it’s much, much harder. You really do need a society that’s prepared to pay for an international movement. (Elaine).

### Capacity building

Most activists reported formal and informal capacity building opportunities in which they were involved. Several emphasised that capacity building is not just about individual skills, but also about relationship-building to hold organisations together and to create a shared culture. Recruitment, local context, and interpersonal relationships were cited as important elements in formal learning programs. Sean emphasised that it is these processes of activism, and ‘participating or engaging’ that are more important for capacity building than fixating on the outcome.

#### Formal capacity building

The PHM’s International People’s Health University (IPHU)^2^ was prominent in comments. Peter discussed the IPHU’s role as being a tool to reach local and young activists and to develop country and region-specific activism. From her experience with IPHU in South Africa, Kate commented on its ability to invest in young people’s learning. She also argued for a greater focus on recruiting and developing the skills of local people and improving post training support:

I’ve had people contacting me and saying well I’ve attended this fantastic IPHU, I’m all inspired and now what?! So how do we harness people?

Angela’s experience with the IPHU in Latin America highlighted how learning was aided by a Freirean [29] popular education approach, which focused the course on the socio-political and cultural context of the place attendees were living in. This meant content resonated personally with them and increased the likelihood of impact.

Rohit explained how the processes of learning were collective in a community run research organisation in India. Students are encouraged to work with three local communities over a period of 12 months and to share and analyse what they experience with the rest of the group – so that ‘learning becomes collectivised’.

#### Informal capacity building

Many activists attributed their capacity development to learning opportunities that arose informally in the context of their civil society work. Ivan described how his training as a medical doctor then led him into working for an NGO and engaging with working class communities and activism:

I was working there in a medical centre of some progressive organisation, [with a] group of progressive doctors. It’s called Doctors for the People, and they [had] set up some medical centres in workers’ neighbourhoods. [T] hat was my first encounter with trade union activism, with local activism, with community activists, and [that] is how I got involved in health activism*.*

Likewise, Emma worked as a young health practitioner in slum and rural areas in the Philippines during the 1980s and consequently joined progressive medical student groups. Samy worked in public hospitals in Egypt in the 1990s during rapid privatisation of the health system while also being active in workers’ rights groups which exposed him to many lessons in how to challenge institutional systems and mobilise people. He spoke of ‘getting people in the street to change the regime’ and ‘increasing the anger within the disadvantaged people’ to ‘change the power structure’ rather than negotiate with it.

In the early 1990’s Kate worked for a community health organisation where ‘a pre-requisite to employment was that you had to have an activist background’. The organisation had a strong political orientation in which they ‘perceived that there were a lot of skills that you gained as an activist that would help you with working in communities’. The organisation’s objectives were developing the capacity of individual workers who were providing health care services and creating an organisational culture of solidarity within the organisation and with marginalised community members including ‘to improve the conditions within which the community lived and to lobby for better services’*.*

#### Organisational culture and reflexivity

The narratives shared by many of our participants pointed to reflexivity as an essential skill for activism, and as a tool for capacity building. Peter stated that reflexivity is about ‘recognising my complicity in the structures that I want to change, and being able to learn about the self and my complicity’ through a ‘process of learning from practice, actively interrogating my practice and my experience’.

The activists described how reflexivity helps to manage tensions between the self and the group which may arise from differing power dynamics and viewpoints, to build relationships and support continual and collective learning. Rohit described how reflexive practice guides his activism and his approach to mentoring younger activists:

But as you do in life, and try to change the world around you, please remember you have to change the world inside you ... And every activist and everybody who comes here knows that you’ve got an inside learning and an outside learning.

Irene discussed why reflexive practice has been important to building her individual capacity, stating that ‘It helps you to adjust because in this activism you need to adjust a lot ... it’s a high relational activity’*.* Irene emphasised the importance of creating spaces within civil society groups which strengthen interpersonal relationships, solidarity, reflection, and learning. She argued that this can lead to accepting and accommodating peoples’ differences and strengths and building a greater diversity of skills:

So, if you put me in a conference hall, I am perfectly at ease. If you put me in a ballroom and you ask me to dance, I may not be, and some other people will. So if you change completely as a setting the strengths of other people can appear, [can become] manifest. And that’s what we did in the group process.

For Irene, such group processes allowed her to step back from her perceived role as ‘leader’, which she found both ‘liberating’ for herself but also that it ‘put a lot of dynamic in the group’.

### Campaigning, advocacy policy dialogue and engaging in governance

#### Linking local, national and global campaigns

Our study participants spoke at length about the importance of, and challenges in, linking local, national, and global campaigns. They voiced concerns that not maintaining links to the local community and grass roots activism would lead to a disconnection with the people they hope to represent in national and global fora. As Samy pointed out, remaining connected to the local is about accountability, ‘If you don’t have constituency - you’re not accountable to anybody, even ethically’*.* Several activists commented on the need to seek a balance between acting locally and globally, but also the difficulty in doing so:

[T]hat’s a balance that’s not always very easy to maintain, and global work, in itself, can suck people into a long-term commitment which often draws them away from their connection to work at the national and local level. (Indra).

A recurring theme from the activists was that local action tends to be ‘working towards a common vision’ with people who in general have the same values, whereas nationally and globally the advocacy is often trying to ‘influence people who are often not coming with the same progressive thinking’. (Kate).

As the global and local become more connected, Michael and Lila point to the necessity to understand how they interact and argue that it is important for activists to work at both levels.

… in a globalised world that struggle, although focused at the local level, it’s going to have to be global as well and that’s … going to require great sophistication, an ability to integrate both in one’s own thinking and strategy and in one’s actions. (Michael).

One of the problems has been that when the policies get changed, all the work you’ve done at the grassroots can get neutralised … So, working on the policies has been more difficult, and the vested interests are very deeply rooted … from the bureaucracy to the politicians to the big corporations. [L] ike those who are working on food … market food prices are spiralling – so you [need to] look at the trade component of it also … what the bilateral and regional trade agreements are doing … [W] orking with the grassroots alone is not enough. (Lila).

This tension between local and global forms of activism emerged as one of the most important ones facing PHM activists, and that, as Michael felt ‘is one in which we don’t do well’. As Indra comments ‘many policies are unfortunately being decided at global centres of power. It’s important to do that, but that has to be tempered with the necessity to continuously link this work with what’s happening in our [local] situations’. (Indra).

#### Reactive campaigns versus proactive

A common theme amongst our activists’ narratives was that the global political economy is becoming more hostile to the HFA goal. This means that campaigns are very often reactive and opposing new developments, giving little scope for activism to be proactive about what developments would be desirable. Elaine discussed an example of reactionary activism. In this instance the government was moving towards privatisation of public assets and threatening to cut health and social services. She describes how a network of public health organisations opposed this:

[We] opposed the privatisation, commercialisation of health services and privatisation of essential social determinants of health like electricity, water and so on … We lost all the federal government funding to the association, because we ran such a successful campaign, I think. I think it was a mark of our success. That was a really important policy, to get universal, publicly funded health care … that was a good campaign but what you realise over the years is it’s just a constant battle to maintain that.

Other activists echoed the reality that their campaigning struggles were constant and that they often felt that they were working hard just to keep still. But they also spoke of continuing on out of their sense of commitment and the solidarity they gain from working with others. All agreed with the sentiment expressed by one that while conducting reactive campaigns ‘At the same time, one should still have and fight for a much more radical vision’. (Michael).

#### Engaging in policy dialogue

The interviewees described how policy dialogue involves challenging vested interests, including commercial interests. As Samy reflected, when ‘you’re challenging interests, don’t be naïve to think that sitting on the same table with a pharmaceutical company is going to lead to a fair game. They are more powerful than you and me’.

Critical to the frustration of campaigning on structural issues was the issue of the centralisation of power and growing global inequities due to the unequal distribution of economic resources.

… the concentration of power and economic power is continuing even 40 years after I started fighting against it, but when we think we have gone forward and made a small victory the other side is making two victories. We have to basically keep up in this rat race … [W] e organise and we have our meetings and we have our plans, but on the other side they also [do the same]. They have the money and therefore are always one step above us. (Nicolas).

However, Lila shared a positive experience of engaging in legal and policy advocacy. While working in a Drug Action Network in the early 1980s, Lila described how women’s groups, health groups, and consumer groups all joined together and through a public hearing in the Supreme Court of India exposed drug companies that were producing harmful contraceptives. Despite the legal experts and money behind the drug companies, after a lengthy battle, not only was this particular drug banned but it led to legislation changes to include a ban on hazardous drugs.

One important lesson Rohit shared was that structural power relationships and inequities that are evident in community work risk being hidden in policy dialogue work. He noted that policy dialogue work requires vigilance and reflection to guard against reinforcing these power inequities:

So you have class, caste, patriarchy, social exclusion. At the community level, it’s stark and you can see. But it’s not as if it doesn’t exist at the district level or at a national level because, in a way, our political systems, our social systems just … replicate themselves at different levels … It’s more subtle at the other levels, you know, like racism or even things like elitism because people are more educated.

Moreover, in order to create a policy dialogue and influence policy, activists often work with government bodies and thus have to balance competing interests. Indra noted that activist work can be:

… with the government [or] against the government. In India, we straddle both. Many of us, including me, are part of government task forces. But at the same time, we retain our independence, and I’m often very, very critical of the government on many issues.

In some cases, the participants reported extreme costs and risks associated with policy dialogue activism, ranging from activists who had been assassinated, injured, arrested, or exiled by oppressive regimes, through to NGOs losing government funding for advocacy.

### Knowledge generation, dissemination and access

The activists collected, generated, and deployed knowledge in numerous ways to challenge neoliberal policies and hegemonic discourses of health and society that PHM understands as creating and sustaining health inequities. Building on Carroll’s [24] framework we look at some of the ways in which the activists deployed counter-hegemonic knowledge practices.

#### Deploying scientific, technical, and legal information

The activists reported the avenues for challenging the system included examining, researching, synthesising, and deploying technical, scientific, and legal information. These efforts were seen in advocacy work, written policy proposals, interactions with government, media and community organisations, academic research and writing, and speaking at local and global conferences and meetings.

As one example in Indra’s interview: an industrial gas leak that caused the death of thousands of people in India in 1984 spurred Indra to join to the People’s Science Movement. This movement focused on researching and educating through science, ‘not just in the context of its potential as a liberator, but also its context of how it’s misused under capitalism, and the kind of social control that you require over science’ (Indra). Questioning how scientific knowledge was being misused and silenced by the Indian government and big industries became central to the group’s activism in developing counter-hegemonic knowledges. Indra explained how the People’s Science Movement employed this strategy across many issues including access to medicines and the pharmaceutical industry, and through a health literacy program used education to mobilise people around issues of health care, water, sanitation and nutrition. Several of these strategies informed the later work of PHM.

Eight participants in this study fall into the category of ‘academic activists’. While recognising their positions within the dominant system as potentially reproducing hegemonic power dynamics, they viewed themselves as able to act as a ‘tool for a broader community’ (Irene) as their positions afforded them the agency and power to elevate the voices of marginalised community members and to disseminate knowledges from the lived experiences of these communities. They often used their social capital and technical skills for creating policy dialogues with people in positions of power and advocating for progressive policies. As Sean noted:

I’ve been able to manage both careers in academia and [as an] open activist … [I] t is possible to manage both things, although there is a risk, and that you have to be prepared to take a risk at the same time.

#### Knowledge as power

When working in the 1970s in Central America in a joint government/NGO program training community leaders, Angela used her position in the community to empower local leaders with legal knowledge that was being withheld from them by their government. Through this example Angela highlights the value of assisting marginalised people to understand their situations through sharing knowledge, and also to demonstrate how knowledge is tied to power relations which in this instance resulted in her having to leave the country.

We taught the people the constitution of (name of country), but it was written in the (local language) and so it was a subversive document. That was my crime, teaching the people the constitution. My crime was having subversive literature … We taught the constitution to local people who are the Justice Department in a little village. They don’t have judges and things like that, but they helped to solve problem [s] locally.

#### Experiential knowledge and reshaping world views

Emma drew on her many years of experience working as a doctor in communities providing health services, training, and popular education programs:

I think always learn from the masses because they will always tell you what their needs are … you have to listen to them. Analyse with them what is happening and together you can come up with a course of action that will really lead towards a positive development.

Many activists emphasised the value of experiential knowledge, and how together the health worker/activist and community members build knowledge and decide on actions.

Ivan’s work in the late 1990s with farmers in the Peasant Movement in the Philippines illustrates the value of collaboration. Ivan assisted the farmers with English, and with work on international issues like trade relations and intellectual property. His Western intellectual assumptions were challenged by working with this group of poor farmers: ‘I learned a lot from them, from the farmers … you discover that actually they have – that the real scientists are those who are in the field’.

Mary argues popular education approaches founded on Paulo Freire’s [29] theories of ‘conscientization’ promote continual and collaborative learning and help to ‘create something new’ which ‘creates the change in society and it can create the change in systems’. Angela elaborated on how she enacted ‘conscientization’ by engaging with local people to make them more aware of the social and political contradictions in their lives and creating a desire to resist oppression.

Some people are not so political and so we have to build a consciousness around those things that we’re working on. But when people are repressed, because they’re defending their water source or trying to stop a mine or a road from being built to get to the mine or whatever, all those kind of things … People get it, even if they’re not political … So, what we’re trying to do now is to build on those things locally and share the experiences across our country.

Furthermore, drawing on lived experience can also bring marginalised communities’ perspectives into mainstream discourse to reshape and challenge dominant world views. Angela described the PHM in Latin America’s decision to incorporate Indigenous knowledges and philosophies into their thinking: ‘the philosophy of the cosmovision of … buen vivir’, as ‘a model that is not exploitative of our planet’.

A benefit of examining the narratives of long-term health activists is being able to comprehend that while activist world views change so do those of the broader society. For example, Nicolas discussed how the concepts *health equity*, *social determinants of health*, and *Health for All* have entered the mainstream consciousness, and the role evidence gathering and knowledge dissemination has played.

What most of us, the senior activists … were already advocating from our empirical knowledge 25 or 30 or more years ago is now becoming more and more mainstream, based on what is called gathering evidence-based information and data.

#### New forms of communication

In their narratives, PHM activists shared their concerns about new forms of communication and collective action. There were different views over whether social media and online activities such as “clicktivism” [30] were a hindrance to activism or whether they provided another useful mode of communication. The merits of social media were viewed differently and largely this reflected a generational difference. Ivan argued the importance of the role of social media in campaigning:

Many older activists were distributing leaflets in the street when they were younger activists, and the equivalent today is tweets on Twitter … It’s just communication. It has a very important role to play whereas, yeah, it was leaflets and flyers, print, newspapers before. Today it’s social media. Tomorrow it will be something else but it’s just communication, and communication is central to activism. Without communication, there is no activism.

Older activists were more sceptical and favoured personal contact and organising, arguing that social media ‘takes away people’s ability to verbalise and vocalise and speak out and go and say it’, and so should only ‘complement what you’re doing but it can’t be the core of what you’re doing’ (Kate).

## Discussion

### The dilemmas of civil society advocacy for health: challenging hegemonic capitalist neoliberalism and individualisation

Many of the dilemmas facing civil society advocacy for Health for All described by this group of long-term health activists reflect the impacts of the changing local and global political, economic and social dynamics over the last 50 years. The activists described these dilemmas as having to grapple with opposing viewpoints, such as whether to focus on single issue health policy reforms or to push for transformation of broader socioeconomic and political structures that condition and constrain health policy options. Such tensions between strategies are not unique to PHM activists, and are described in other literature on community development/mobilisation and social movement building [31, 32]. Activities relating to health were positioned as having more urgency however, because of their direct impact on individuals.

The changes in social organisation during late modernity with increased individualisation and the dominance of capitalist neoliberal economic and political systems has contributed to unequal distributions of resources and power between and within countries [33]. It is these inequities that motivate the activists to mobilise. Thus the strategies for building social movements and the capacity of civil society to advocate for health equity are deeply interlinked with challenges to the dominant neoliberal economic systems and the absence of effective global governance structures to regulate in the interests of the health of people and the planet.

In this section we introduce the concept of late modern individualisation and outline why a socio-political analysis of these global trends is important to understanding the challenges faced by groups mobilising collective action and building social movements for Health for All. The discussion then examines central dilemmas facing current civil society health activism raised by the activists and how they navigate these systemic challenges to work towards social change to improve health.

Individualisation theory suggests that two factors characterise our late modern era: ‘choice and reflexivity in identity and the privatisation of social and political problems to an individual level’ [34]. Individualisation represents structural changes between the individual and society, in which the individual takes precedence over society or social communities [35] and social reproduction is individually generated [34]. For example, identity once tied to class, community, culture, or family tradition, has moved from a ‘given’ into a ‘task’, and a task in which individuals have more freedom to choose, but are also more responsible for enacting the set of tasks and norms associated with certain identities (e.g. gendered norms, such as boys don’t wear skirts, and women are responsible for domestic work) [36]. This does not mean that these categories and identities are obsolete or that the influence of these structures on society has diminished, rather the relationship between them and the individual has shifted. Beck and Giddens view this freedom and the individual’s ability to act as central to late modernity. However, Bauman highlights the Western assumptions taken for granted in this position and argues while repression may have lessened, this freedom is to some extent illusionary and certainly not universally experienced [37], referring to individualisation as the uneven ‘redistribution of freedoms’ [36]. While individualisation may have created more freedoms of choice and identity for certain groups, in the current hegemonic neoliberal era only the very privileged have significantly benefited. Instead, for many, individual responsibility for basic necessities has increased, and the responsibility of the collective to the individual has decreased, as national and global elites have become disembedded from national and local responsibilities. This argument has been central to PHM’s HFA efforts, and as Rohit cautioned above, systems of power and discrimination while often less overt in global policy and advocacy settings can be just as, if not more, impactful on communities and individuals, while accountability is more difficult to trace back to global actors.

In rather stark (or dialectical) contrast, civil society groups such as the PHM are specifically concerned with building a social solidarity that has been weakened through neoliberal policies and discourses which encourage people to seek individual solutions to their problems [19, 35]. The study findings illustrate how within the many local and global civil society communities in which the activists worked, they and their social movement comrades have been growing and nurturing alternative approaches to structuring society and improving health and wellbeing.

### Individualisation of civil society: challenging the de-politicisation of civil society spaces

Our findings highlight growing concerns over the de-politicisation of individuals and groups in civil society. The activists positioned a progressive ideological foundation as being essential to the advocacy for health improvements and health equity, and felt this was lacking in many NGOs working in the field of health. NGOs have come to dominate the civil society space, receiving the majority of funding from government and international bodies, in particular to play technical roles or provide services [38]. Edwards [4], echoing the sentiments of the activists in this study, argues that post-1990 civil society has been co-opted by governments and aid agencies with ‘the replacement of civil society by a set of narrower concepts that are easier to operationalise such as the “third-sector”, the “non-profit sector”, and the “social economy”’ [4]. Debates about the economic role of civil society dominate over their cultural and political significance, especially as states continue to retreat from their social obligations to provide essential services [4]. Kapilashrami and McPake [39] critique this funding environment which privileges business-like and large international NGOs, arguing it is impacting civil society more broadly through a loss of capacity, advocacy and movement building skills and thus the ability to respond effectively to health and equity issues at the local and global. In contrast informal and formal capacity building of individuals and groups was described as central to the work of the PHM and other activists’ groups in the activist narratives.

The activists in this study felt many employees of NGOs lack a political or ideological foundation, including of HFA, and are more often motivated by career advancement. Along with the reasons stated above, they highlighted how this is detrimental to the functioning of civil society, which without an ideological focus on challenging political and economic structures that lead to inequities is not only at risk of excluding citizens voice but reproducing and increasing inequities. These trends reflect a neoliberal individualisation of the civil society sector, in which workers are disembedded from the collective, organisations are encouraged to be competitive rather than collaborative, programmes and projects are siloed, and activities are tied primarily to an economic value.

This narrowing civil society environment affected the interviewees’ view of their own work. In the narratives there is a strong dialectical tension between campaigns which deal with specific issues (often cited was the campaign for affordable drugs which had introduced many of the interviewees to health activism) and working on the structural underpinnings of the single issues (e.g. neoliberalism, colonialism). The PHM’s niche is that it pays particular attention to campaigning on structural issues. Focusing on single issues risks ignoring inequitable underpinnings of national and global economies. Labonté [40] questions whether it is possible to link issue-specific campaigning and organising to system-oriented change:

Health activists are left with a paradox. The simple message of a particular issue is good grist for campaigning. But without being anchored in a deeper yet simplified statement on societal structures of power, it lacks the ability to build a broad-based civil society movement for the type of society that might transform the escalations in wealth inequalities.

Under the theme on ‘Civil society organisations – building social movements or businesses?’, the activists described how this trend has impeded capacity building of civil society to grow social movements, including HFA. In response, some of the activities of PHM, and especially the International People’s Health University short courses, are designed to politicise NGO and other health and social service workers to encourage them to go beyond the provision of curative services to extend to prevention and advocacy for structural changes to economic and political systems.

We found this ongoing tension for health activists has been made more difficult by funding bodies and governments predominantly channelling funds to NGOs working on service provision or single-issue projects rather than broader projects critiquing the political and economic status quo and arguing for a fairer system that focuses on health.

### Working at the global while representing the local

Linked to the concerns raised above is the centralisation of power in well-funded international NGOs engaging with global elites [41]. This centralisation of power and decision making runs the risk of NGOs, specifically, and civil society social movements more generally, being disconnected from the populations they are often said to be representing and co-opted by vested interests. This is in direct conflict with the objectives of HFA which includes a commitment to citizen participation in decision making about individuals and their communities health to improve population health [19, 21].

Kapilashrami and O’Brien [41] highlight one consequence of this shift in power to international NGOs. They investigated the large AIDS NGO ‘industry’ and raised concerns that a ‘de-politicisation of critical voices’ has occurred and the increasing technical discourses of large NGOs have diverted attention away from advocacy work ‘in order to fit in with formalised models and frameworks of mainstream development’.

The interviewees were acutely aware of the need for a progressive social movement voice in global fora, but also of the tense balancing act involved, and the dangers of investing too heavily in either the global or local. Policy dialogue was seen to complement other forms of activism, such as working in community health clinics. There was, however, a caution raised of activists seeing community work as more ‘authentic’ than engaging in policy dialogue, especially at global scales. As Indra pointed out policy decisions are often made at the ‘global centres of power’ where the neoliberal economic agenda favours individual global elites over the majority of the world’s population. Bodini et al. [21] argue ‘a critical priority for the HFA movement is therefore to develop the capacity to act as a global social movement’ including to provide a voice in global policy settings. A central goal of PHM efforts is to influence policy at the World Health Organisation (WHO), partly through its WHO Watch program that involves bringing new and younger activists to Geneva for policy analysis and lobbying purposes. Much of this work also formed the texts of the *Global Health Watches* [11–15].

The challenge in this global/local balancing act, as our larger study noted, lies in ‘addressing the local and immediate issues in ways which also contribute to redressing the larger scale and longer term dimensions’ [21]. As argued by the activists and a previous study of community health activism [42], the local and global are intrinsically connected in the current globalised era and thus, despite the challenges of globalisation, the struggle for health equity must work at all levels of society.

### Navigating neoliberal individualisation and possibilities for social movement building

Since the 2000s reactions to hegemonic capitalist neoliberalism have brought a resurgence of interest in social issues campaigning and a continuing interest in the Health for All movement [43]. As our larger research study found, there have been few progressive policies that have been implemented without the advocacy and mobilising efforts of civil society activists and organisations [21]. In recent years civil society organisations have also come to play a more prominent role in global health governance [41, 44, 45]. The narratives demonstrate some of the ways in which long-term health activists have negotiated and navigated late modernity and neoliberal individualisation processes to continue working with and against dominant systems to improve health.

Through their global-local civil society networks the activists highlighted the possibilities that late modernity and globalisation offer to civil society organisations and advocacy for health, whilst remaining vigilant in resisting the forces discussed above. Knowledge generation and access, for example, were regarded by activists as powerful tools by which to connect and empower groups living in disadvantaged circumstances, potentially leading to social change that creates a ‘globalisation of norms’ [46], and evidenced by changes in international rules, such as United Nations conventions and declarations [47]. Wills and Pearce argue time-space distanciation defines globalisation and through various communication technologies social relationships are able to ‘stretch across the world, for people in distant places to link with each other in the here and now and for what happens in one space/place to impact on people in other places’ [48]. Despite uncertainties in our activists’ narratives about the internet and social media, these modes of communication have assisted civil society groups and social movements to connect and share knowledge (e.g. linking marginalised LGBTIQ individuals to support groups and social movements [49]). Social media has in recent times been crucial to making activism visual to audiences across the world (e.g. Black Lives Matter protests in the US) and catalysing real life social activism [50]. Funnell’s [50] argument is shared by many of the participants in this study, but with the *caveat* that, while the internet and technology can enhance the effectiveness of activism, physical commitment and participation is needed to underpin its success.

Health for All has always consisted of an outward global looking approach to addressing health equity. However, since its origins the social landscape has changed dramatically, including increased globalisation and social connectedness which has resulted in health activists sharing common experiences in different settings across the world [21]. As individuals can call on the knowledge of ‘expert systems’ [51] to assist in understanding their localised and personal situations, there are more opportunities to build an inclusive global strategy and social movement for HFA.

Reflexivity was a key theme in the narratives for building the capacity of individuals and organisations in civil society. Beck, Giddens and Lash’s [52] concept of ‘reflexive modernisation’ highlights how individuals have become reflexive agents, in which an individual is granted more agency, but also increased responsibility for their identity, actions and relations with other [34]. Late modernity has been driven by a revaluation of modernisation, where the social cohesion of categories such as class and the family unit once dominated over individualism. As reflexive agents, individuals in late modernity are no longer embedded in industrial society’s predetermined social categories and identities, and it is during this process of removal that individuals become reflexive in their relationship to other individuals, social structures, and institutions [53]. Giddens calls individualisation ‘the reflexive project of the self’ [54] and Rasborg [35] highlights how individualisation refers to the fact that individuals increasingly take themselves as a point of reference in late modernity and (neoliberal) capitalist structures. This points to individualisation being experienced as socio-culturally specific, and therefore experienced differently by different groups [34].

This form of reflexivity is also evident in the narratives. It is discussed as a key tool for making sense of one’s own position in the social world, sharing those reflections with others, and encouraging others to also be reflexive, all with the intent of initiating social change. This process can act as a catalyst for personal and wider transformation:

… we are all engaged in producing the world. Reflexivity enables us to place ourselves actively within this process ... By actively and critically reflecting on the world and our place within it, we are more able to act in creative, constructive ways that challenge oppressive power relations rather than reinforce them [55].

Maxey suggests reflexivity in activism is ‘something that can usefully be employed to help counter social exclusion at all levels’ [55]. As the larger PHM study concluded:

A significant element of cultural development for activist organisations is the modelling of transgression. The norms of the establishment have a powerful hold on all of us, activists old and new. The act of transgression is part of shaping a collective identity which stands apart from those established norms, in aspiration if not always in practice [21].

Reflexivity, then, is a tool for both transgression and social change in the current era of global capitalist neoliberalism. Individualisation creates spaces for reflexive practice, and is not necessarily opposed to community and collective identities but rather, and more dialectically, as Rasborg [35] suggests expresses a new (reflexive) way of relating to communities. Individualisation has allowed disruption of traditional and sometimes oppressive social customs and norms to be replaced with newer forms [53]. The examples of capacity building and formal learning in the narratives demonstrate how this is possible through building the knowledge of individuals that they can take back into their own worlds and workplaces, while also bringing them into contact with broader civil society circles and social movements. However, as noted above, while positive and transformational for some, in the current hegemonic capitalist neoliberal era, individualisation is so often deployed by elites and governments through a narrow agenda of ‘individual responsibility’, reinforcing inequities. Therefore, we do not argue that individualisation and reflexivity replace progressive social movements (such as trade unions) as a force for social change, rather that there may be benefits in exploring the new possibilities they offer to civil society activism and the goal of Health for All.

## Conclusion

This study has examined the experiences of long-term civil society health activists through the prism of hegemonic capitalist neoliberalism and neoliberal individualisation to better understand the structural factors which have created tensions for the activists in their advocacy for Health for All. The narratives also revealed strategies that make for effective advocacy for improving the social determinants of health and health equity. Despite the pervasive neoliberal policies which have seen both individuals and groups disembedded from the collective and worked to undermine civil society activism, the narratives illustrate how the long-term activists’ have been challenging these processes and creating social change to support Health for All. Their strategies for building and sustaining civil society were orientated towards building collective solidarity and creating networks between individuals and groups, local and global. At the same time, however, activists recognise the reproduction and growth in inequities as power and wealth have become more centralised and unequally distributed. With growing concern over the depoliticisation of civil society and the shrinking spaces for democratic participation, this study of activists’ practical experiences suggests that civil society activism for health must constantly be engaged in socio-political and reflexive analysis in order to advocate for the structural conditions for health equity. In doing so, their work of necessity entails activism beyond single issues to incorporate and advance arguments supporting deeper and more fundamental forms of structural change.

## Data Availability

N/A

## Abbreviations

PHM: People’s Health Movement
HFA: Health for All
NGO: Non-governmental organisation
IPHU: International People’s Health University
WHO: World Health Organisation

## Footnotes

1 To delve further into the work of the PHM, the special issue of ‘Saúde em Debate’ (2020) showcases the vast activities of the country circles and members of the PHM. See http://revista.saudeemdebate.org.br/sed/issue/view/33

2 For more information about PHM’s International People’s Health University see https://www.iphu.org/.
